# The use of theory of planned behavior to systemically study the integrative-qualitative intentional behavior in Romanian preschool education with network analysis

**DOI:** 10.3389/fpsyg.2022.1017011

**Published:** 2023-01-09

**Authors:** Dana Rad, Adela Redeș, Alina Roman, Anca Egerău, Raul Lile, Edgar Demeter, Tiberiu Dughi, Sonia Ignat, Evelina Balaș, Roxana Maier, Csaba Kiss, Vasile Mărineanu, Mușata Bocoș, Graziella Corina Bâtcă-Dumitru, Lavinia Denisia Cuc, Gabriela Vancu, Gavril Rad, Roxana Chiș

**Affiliations:** ^1^Faculty of Educational Sciences, Psychology and Social Sciences, Center of Research Development and Innovation in Psychology, Aurel Vlaicu University of Arad, Arad, Romania; ^2^Academia Oamenilor de Știință din Romania, Bucharest, Romania; ^3^Faculty of Psychology and Educational Sciences, Hyperion University of Bucharest, Bucharest, Romania; ^4^Department of Psychology, University of Bucharest, Bucharest, Romania; ^5^Faculty of Psychology and Educational Sciences, Babeș-Bolyai University, Cluj-Napoca, Romania; ^6^Faculty of Accounting and Management Informatics, Department of Accounting and Audit, Bucharest University of Economic Studies, Bucharest, Romania; ^7^Faculty of Economics, Aurel Vlaicu University of Arad, Arad, Romania

**Keywords:** integrative-qualitative intentional behavior, preschool teachers, early childhood education and care, SDG4.2, assessment validity, factor structure

## Abstract

Predicting preschool teachers’ intention to adopt qualitative and inclusive early childhood intentional behaviors represents an important research field. The objective of this research is first to develop and validate a scale to assess the integrative-qualitative intentional behavior (IQIB-ECEC) of preschool teachers in order to achieve SDG4.2’s objective of ensuring that all children have access to high-quality pre-primary education and then to systemically analyze the relationship between variables with Network Analysis. The theory of planned behavior (TPB) describes key individual beliefs (attitudes, subjective norms, and perceived behavior control) that affect people’s intentions to engage in a certain conduct and has previously been used with success in evaluating people’s intentions to adopt a certain behavior. This research represents one of the first Romanian attempts to use the theory of planned behavior to study the Integrative-Qualitative Intentional Behavior in Romanian Preschool Education and systemically analyze results with Network Analysis approach. This study used a randomized 300 Romanian preschool teachers enrolled in a National Training Program entitled Qualitative and Inclusive Early Childhood Education managed by the Romanian Educational Ministry. Data were collected *via* an online questionnaire. The scale validation followed a confirmatory factor analysis (CFA). The fitting of the IQIB-ECEC 19-item scale showed that all coefficients CFI (0.942), TLI (0.920), SRMR (0.0452), and RMSEA (0.0451) bring strong evidence in the favor of the statistical validity of the scale. The final IQIB-ECEC 19 items and 8 factors scale obtained a Cronbach’s alpha of 0.77. The systemic Network Analysis approach was used in interpreting data. The centrality of the network model was further investigated and the clustering coefficients index were calculated. According to the results, perceived power/control beliefs and behavioral intention were detected as the most important dimensions, whereas behavioral beliefs were less important. These findings were discussed in terms of their theoretical and practical significance.

## 1. Introduction

Any society’s long-term prosperity will depend on its capacity to advance the well-being and health of the upcoming generations. The youngsters of today will grow up to be the parents, workers, and citizens of tomorrow. Our wise investments in families and children will pay off in the form of lifetime productivity and civic involvement from the next generation. Child development serves as a foundation for both societal and economic progress since competent children provide the foundation for a successful and sustainable society.

Making sure nobody is left behind, particularly the most vulnerable individuals across all goals, is the core objective of equality. Extreme poverty, insurgency, violence between communities, and other issues have all seriously impeded development in numerous nations. Compared to their wealthy contemporaries, children from low-income homes are more likely to drop out of school. There are still significant differences between rural and urban places.

This decisive engagement must be made a reality for all children, youth, and adults in the years leading up to 2030, regardless of where they reside or the challenges they encounter. Equity indices include personal factors like gender, region, ethnicity, language, disability status, and child labor involvement as well as family traits like parents’ socioeconomic status, wealth, or educational attainment.

As part of the Sustainable Development Goal 4 (SDG4) targets, nations are required to keep analyzing these statistics in order to improve their quality in order to monitor advancement over time. Policies and rules must take into account educational system inputs in addition to results and outputs in order to successfully monitor some SDG 4 targets. In general, inputs are parts of the education system over which the government has relatively direct influence.

Target 4.2 aims to ensure that all girls and boys have access to high-quality early childhood development and care services by 2030 in order to be prepared for elementary school. Pre-primary education is also an objective of this target. More specifically, access equality to high-quality care and education services is addressed by indicator 4.2.1, which breaks down by gender the percentage of children under five who are on track in terms of their health, learning, and psychological well-being. Because many youngsters do not enroll in full-time educational programs throughout their formative years, the level of their exposure to learning contexts outside the family will vary. It is hard to determine whether this objective has been accomplished because the indicator only counts the proportion of kids who get structured learning, not the program’s intensity. Through a range of global development courses, “Education for All” has gained popularity since 1990. It was designated SDG 4 when the Sustainable Development Goals (SDGs) were initially created because it was seen as being extremely important. Education is seen as a tool for fostering peace, establishing nations, and promoting sustainable development. Learning specific abilities, like reading, writing, or counting, increases a child’s or adolescent’s likelihood of success in life.

The importance of education in promoting sustainable development may benefit not only underdeveloped nations, but also the entire world. The main goal of Sustainable Development Goal 4 (SDG 4) is to guarantee that all people have access to a good education that will enhance their quality of life and the future of their society. Target 4.2’s major goal is to guarantee that, by the year 2030, all children gain access to high-quality early childhood education to prepare them for elementary level.

## 2. Theoretical framework—theory of planned behavior

The theory of planned behavior (TPB) outlines important individual beliefs: attitudes, subjective standards, and perceived behavior control that have an impact on people’s intentions to engage in specific behaviors. TPB has been effectively used in individual behavioral change interventions, and as a result, it has served as the basis for numerous research examining teachers’ intentions to advance inclusive education. However, little effort was made to integrate these findings into practice (Opoku et al., 2021; Rad et al., 2022a,b).

The Theory of Reasoned Action (TRA) was renamed the Theory of Planned Behavior (TPB) in 1980 in order to foresee a person’s intention to engage in a behavior at a certain time and place. The most crucial component of this paradigm is behavioral intent, which is affected by beliefs about the likelihood that a certain course of action would produce the desired results as well as a personal evaluation of the benefits and drawbacks of those results.

According to the TPB, both motivation (intention) and ability play a role in behavioral success (behavioral control). The behavioral, normative, and control forms of beliefs are distinguished. The TPB consists of six constructs that collectively represent an individual’s actual level of control over a behavior.

1. Attitudes: The concept refers to how positively or negatively a person regards the activity of interest. It necessitates considering how actions will influence outcomes.
2. Behavioral intention: This relates to the motivations behind a certain activity; the stronger one’s desire is to engage in a behavior, the more probable it is that they will do so.
3. Subjective norms: This concept refers to whether or not the majority of people believe a particular behavior to be acceptable or objectionable. It has to do with whether peers and close friends think the person should engage in the habit, or whether there is social pressure to do or not to execute a specific conduct. Subjective norm is mostly made up of compliance desires and normative ideas.
4. Social norms: The recognized codes of behavior within a community or larger cultural context are referred to by this word. Social norms are viewed as normative or standard among a group of people.
5. Perceived power: The concept refers to how something is perceived as potentially assisting or impeding the performance of an action. According to perceived power, it is believed that each of those factors may be controlled by a person’s actions to some level.
6. Perceived behavioral control refers to a person’s perception of how simple or challenging it is to engage in the intended activity. Because perceived behavioral control varies between contexts and acts, a person’s opinions on behavioral control vary depending on the situation.

Despite consistently producing results over time, the TPB has been criticized for its primary shortcomings, which include the following: while it does take into account normative influences, it still does not take into account economic and environmental elements that might affect a person’s intent to engage in a conduct, such as fear, danger, emotions, or prior experience. In addition, it presumes that the person, regardless of purpose, has access to the opportunities and resources required to successfully carry out the required conduct. TPB considers that conduct is the result of a linear decision-making process rather than acknowledging that behavior may change over time.

According to the TPB, one’s intentions and views of their ability to regulate their conduct can directly predict their behavior (Ajzen, 1991). Additionally, research indicates that intentions are a secondary mechanism *via* which attitudes, perceived behavioral control, and subjective norms influence behavior (Ajzen, 1991; Ajzen, 2020).

Even while this study suggests that the TPB may be integrated in educational settings (Patterson, 2001; Stanec, 2009; MacFarlane and Woolfson, 2013; Heuer and Kolvereid, 2014; Cooper et al., 2016; Burns et al., 2018; Dunn et al., 2018; Bornschlegl et al., 2021) and used to explain integrative-qualitative conduct in preschool settings, it also emphasizes the need for more qualitative research on the beliefs that surround this behavior in early childhood education and care.

This study served as the foundational stage for subsequent investigation into the applicability of a TPB-guided framework to comprehend and address integrative-qualitative behavior in preschool instruction. The main goal of this theory-based research was to qualitatively examine attitudes, subjective norms, and perceived behavioral control connected to the integrative-qualitative behavior among preschool education using both a deductive and an inductive analytical procedure in order to design a valid a reliable scale for further assessing the integrative-qualitative intentional behavior of preschool teachers with network analysis (NA).

We are relying on this scale when further assessing early education integrative-qualitative intentional behavior of preschool teachers in order to design training programs based on nudges and boosts to empower preschool teachers to sustainably implement an integrative-qualitative intentional behavior at work.

## 3. Current research

The objective of this research is first to develop and validate a scale to assess the integrative-qualitative intentional behavior (IQIB-ECEC) of preschool teachers in order to achieve SDG4.2’s objective of ensuring that all children have access to high-quality pre-primary education and then to systemically analyze the relationship between variables with Network Analysis. Thus, the research first aimed to develop a valid and reliable scale to measure preschool teachers’ intentional integrative-qualitative behavior based on theory of planned behavior methodology in order to further assess with Network Analysis the intentional integrative and qualitative behavioral pattern of 300 Romanian preschool teachers. Based on our previous scoping review that clearly indicated that there is a lack of assessing both qualitative and inclusive behaviors in early education (Rad et al., 2022a), we have proposed the IQIB-ECEC scale that will further analyze preschool teachers’ behavior with Network Analysis approach. Network analysis (NA) is a set of integrated techniques used to delineate relations among factors and to analyze the structures that emerge from the recurrence of these relations. The use of NA in psychological scale assessment has previously been successful (Suwartono and Bintamur, 2019).

Another important reason for choosing the theory of planned behavior as theoretical framework is that further in this research project our team proposed an intervention program for preschool teachers designed to enhance actual integrative-qualitative behaviors and particularly we were interested in how we can indirectly attain the desired output, but still controlling for individual factors as identified by Ajzen. By following this methodology, we can further instill self-assessment competencies of preschool teachers in their integrative-qualitative actual behavior based on reflective practice and this newly self-assessment competency in the behavioral domain can further positively affect through cognitive positive transfer processes other educational behaviors related to inclusivity and quality in preschool education.

The basis for the item’s generation was the Ajzen methodology and all 24 items were adapted to qualitative-inclusive behaviors in early education for teachers. The 24 items of IQIB-ECEC were measured on a 5-point Likert scale. Utilizing item analysis, confirmatory factor analysis, Cronbach’s alpha, CFI, TLI, SRMR, and RMSEA fit indices, we tested the validity and reliability of the IQIB-ECEC scale.

Further, we have investigated the relations between all scales’ factors with network analysis in order to explore how to further instill intentional qualitative and inclusive behaviors in Romanian preschool teachers.

This study aimed to reveal the pattern network structure of the 8 dimensions of intentional qualitative and inclusive behaviors in Romanian preschool teachers. Network analysis was applied to the 8 dimensions to define the strong and weak 138 connections in the network, to determine the intensity of interaction in the network, and 139 to reveal the roles of the variables in the network. JASP (Version 0.14; Computer 140 software) was used for the structural determination and visualization of the relationships 141 between variables in the analysis (28).

## 4. Research methodology

### 4.1. Participants

The research sample consisted of 300 educators from West Romania’s 15 counties, of whom all were female. Participants were selected from a project-based national preschool teacher training program, which started in Romania in the spring of 2021 during the COVID-19 pandemic, based on their availability and consent to participate in this online research. The Romanian Ministry of Education has started a national program entitled Qualitative and Inclusive Early Childhood Education for a total number of 2,000 preschool instructors, out of which 700 preschool instructors from West Romania were managed by Aurel Vlaicu University of Arad’s team. After inviting all 700 participants to take part in our online research, 300 valid online responses were returned. In order to collect responses, a Google Form questionnaire of 24 items was created, rated on a typical Likert scale from 1 to 5. Three additional questions were added, that of location, age, and years of previous work experience in preschool education.

Regarding the age of our respondents, the reported age range was between 22 years old and 63 years old, with an average mean of 41 years. As for work experience with preschool children, the range was between 0 and 44 years of previous experience with an average mean of 18 years of work experience.

### 4.2. Instrument

The basis for the item’s generation was the Ajzen methodology and all 24 items were adapted to qualitative-inclusive behaviors in early education for teachers, from former seminal research papers (Ajzen, 1991; Czerniak and Lumpe, 1996; Harland et al., 1999; Hagger et al., 2002; Heath and Gifford, 2002; Francis et al., 2004; Johnson and Hall, 2005; Arnold et al., 2006; Darker and French, 2009; Schomerus et al., 2009; Kortteisto et al., 2010; Lee et al., 2010; Poulter and McKenna, 2010; Ajzen et al., 2011; Ajzen, 2011a; Ajzen, 2011b; González et al., 2012; Greaves et al., 2013; Sun et al., 2015; Hadadgar et al., 2016; Newham et al., 2016; Teo et al., 2016; Qi and Ploeger, 2019; Liu et al., 2021; Pang et al., 2021).

Following an expert small group online meeting for analyzing existent questionnaires based on TPB that have investigated intentional behaviors in educational settings, our research team decided to purposely design an 8 dimensions scale with 24 items (3 items per dimension as recommended by the TPB scale design methodology). The 8 dimensions were: D1. Actual behavior (3 items), D2. Attitudes toward the behavior (3 items), D3. Behavioral beliefs (3 items), D4. Subjective norm (3 items), D5. Social norms/Normative beliefs (3 items), D6. Perceived power/Control beliefs (3 items), D7. Perceived behavioral control (3 items), and D8. Behavioral intention (3 items). After the items’ grammatical and contextual structure was selected, all 24 items (3 items per dimension/factor) were adapted according to the Romanian national characteristics in preschool education.

The IQIB-ECEC questionnaire that was completed by the preschool teachers, during November–December 2021, consisted thus of 24 items (8 dimensions), rated on a 5-point Likert scale, ranging from 1 strongly disagree to 5 strongly agree, and three open-ended questions (location in terms of county district, age, and previous work experience in preschool education).

## 5. Results

This section will further present the statistical validation of the IQIB-ECEC questionnaire and the systemically analysis of all relationships between model’s variable with Network Analysis. Since we have followed Ajzen’s methodology (Ajzen, 1991; Ajzen, 2011a; Ajzen, 2011b) for designing the theory of planned behavior with 8 factors, we have further addressed the statistical validation of the scale based on already established factors, with a confirmatory factor analysis technique.

### 5.1. IQIB-ECEC scale statistical validation

Before running the correlation analysis, we firstly looked at means obtained on all purposely designed 8 dimensions of IQIB-ECEC 24-item scale, with the intention of getting a first view of how the data are presenting itself from a descriptive point of view.

**Figure 1:**
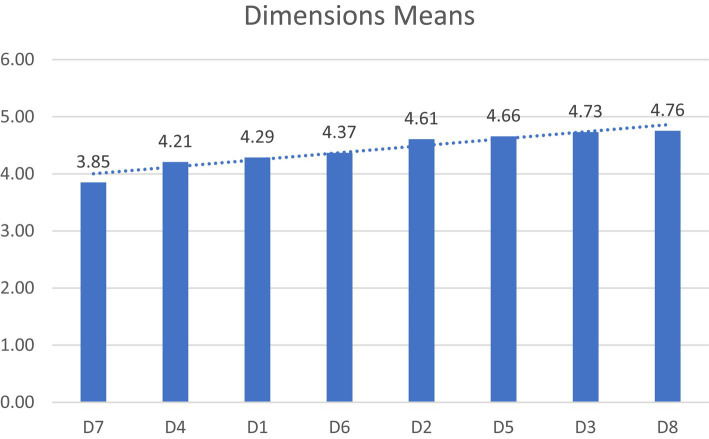
Dimensions means of the initial 24 items IQIB-ECEC online questionnaire.

Looking at the means we have registered on all investigated dimensions, we can observe the interesting dynamics of the proposed TPB variables (Figure 1). On the dimension of D7. Perceived behavioral control, we find the lowest average m = 3.85, we are somewhat in front of a phenomenon of metaphorical abandonment of integrative-qualitative intentional behavior in front of a behavioral constraint felt by our representative sample of 300 ECEC professionals from Romania. The lack of control over one’s own behavior seems to paralyze the whole mechanism of adopting the principles of equity and inclusion in preschool institutions. This aspect is somewhat offset by the higher average obtained on the dimension D8. Behavioral intention, m = 4.76, which reflects the openness of professionals to embrace the behaviors of inclusion and provision of qualitative services to all children.

**Figure 2:**
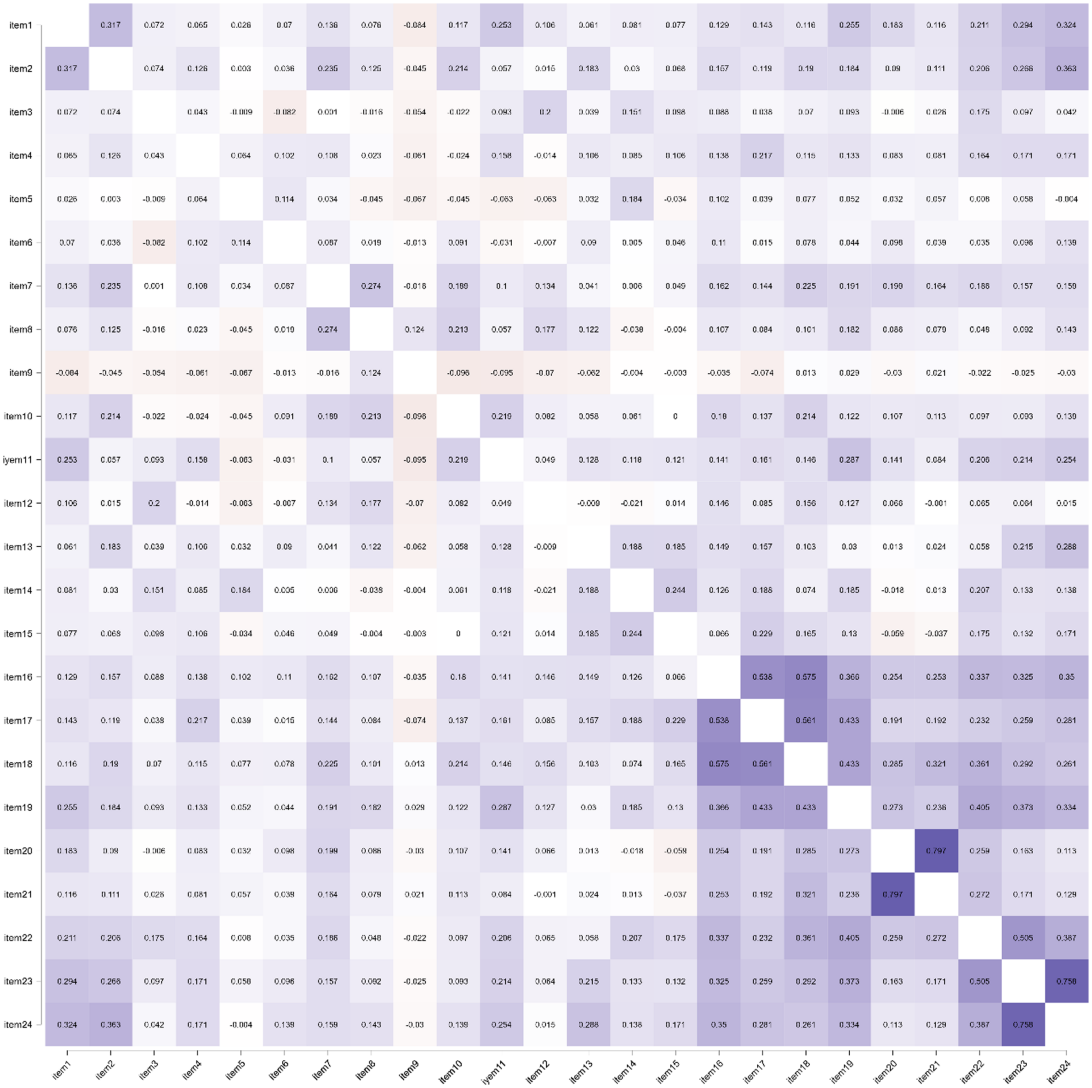
Correlations heat map of the IQIB-ECEC 24-item scale.

We have then tested the correlation of each item with the scale and the inter-correlation between items for all 24 items. In Figure 2, we are presenting the correlations heat map of the IQIB-ECEC 24-item scale, utilizing JASP software (version 0.16.3.0).

Confirmatory factor analysis (CFA) is a statistical technique used to confirm the factor structure of a series of observed variables. CFA enables researchers to test the hypothesis that there is a link between observable variables and their underlying latent components (Harrington, 2009; Brown and Moore, 2012). CFA is employed to examine a hypothesis that has already been proposed. It makes an *a priori* model of the target construct’s internal structure and evaluates how well it fits the available data (Marsh et al., 2009). The fit between the postulated CFA model and the observed data is assessed using several fit statistics. Researchers use these indices to check whether their model adequately represents the data by reviewing acknowledged criteria (Fokkema and Greiff, 2017).

The confirmatory factor analysis was performed in Jamovi, selecting the following items for each dimension (Factor):

- Factor 1 (D1. Actual behavior): item 1 and item 2,
- Factor 2 (D2. Attitudes toward the behavior): item 5 and item 6,
- Factor 3 (D3. Behavioral beliefs): item 8 and item 9,
- Factor 4 (D4. Subjective norm): item 10 and item 11,
- Factor 5 (D5. Normative beliefs): item 13, item 14, and item 15,
- Factor 6 (D6. Perceived power/Control beliefs): item 16, item 17, and item 18,
- Factor 7 (D7. Perceived behavioral control): item 20 and item 21,
- Factor 8 (D8. Behavioral intention): item 22, item 23, and item 24.

Results obtained for CFA in terms of factor loadings after dropping items that had negative loadings or did not reach the inclusion criteria: item 3, item 4, item 9, item 12, and item 19, for purifying the constructs, are presented in Table 1. Scale purification represents the process of eliminating items from multi-item scales (Wieland et al., 2017).

**Table 1:** Confirmatory factor analysis results for IQIB-ECEC 19-item scale.

95% confidence interval								
Factor	Indicator	Estimate	SE	Lower	Upper	Z	*p*	Stand. estimate
Factor 1: Actual behavior	Item 1	0.265	0.0358	0.1946	0.335	7.40	< 0.001	0.562
	Item 2	0.239	0.0323	0.1760	0.302	7.42	< 0.001	0.564
Factor 2: Attitudes toward the behavior	Item 5	0.221	0.1019	0.0211	0.420	2.17	0.030	0.358
	Item 6	0.224	0.1049	0.0181	0.429	2.13	0.033	0.318
Factor 3: Behavioral beliefs	Item 7	0.537	0.0951	0.3509	0.724	5.65	< 0.001	0.682
	Item 8	0.192	0.0407	0.1124	0.272	4.72	< 0.001	0.401
Factor 4: Subjective norm	Item 10	0.306	0.0629	0.1829	0.429	4.87	< 0.001	0.454
	Item 11	0.345	0.0692	0.2090	0.480	4.98	< 0.001	0.483
Factor 5: Normative beliefs	Item 13	0.202	0.0369	0.1300	0.274	5.49	< 0.001	0.454
	Item 14	0.334	0.0626	0.2112	0.457	5.33	< 0.001	0.456
	Item 15	0.261	0.0488	0.1655	0.357	5.35	< 0.001	0.451
Factor 6: Control beliefs	Item 16	0.572	0.0423	0.4895	0.655	13.54	< 0.001	0.752
	Item 17	0.585	0.0459	0.4948	0.675	12.74	< 0.001	0.713
	Item 18	0.623	0.0443	0.5360	0.709	14.07	< 0.001	0.776
Factor 7: Perceived behavioral control	Item 20	1.071	0.0752	0.9232	1.218	14.24	< 0.001	0.917
	Item 21	1.001	0.0734	0.8569	1.145	13.64	< 0.001	0.869
Factor 8: Behavioral intention	Item 22	0.348	0.0373	0.2749	0.421	9.33	< 0.001	0.538
	Item 23	0.333	0.0191	0.2956	0.371	17.40	< 0.001	0.880
	Item 24	0.325	0.0195	0.2873	0.364	16.72	< 0.001	0.853

For the IQIB-ECEC final 19-item scale, Bartlett’s test of sphericity reported a χ^2^ of 200 with df (124) at a *p* < 0.001.

Regarding the fitting of the IQIB-ECEC 19-item scale, all coefficients CFI (0.942) and TLI (0.920), results supported by literature (Xia and Yang, 2019), SRMR (0.0452), and RMSEA (0.0451) results supported by literature (Kenny and McCoach, 2003; Kenny et al., 2015) depicted in Table 2, bring strong evidence in the favor of the statistical validity of the scale.

**Table 2:** Fit results for IQIB-ECEC 19-item scale.

RMSEA 90% CI					
CFI	TLI	SRMR	RMSEA	Lower	Upper
0.942	0.920	0.0452	0.0451	0.0332	0.0564

Finally, with the remaining items, we have performed an internal consistency reliability analysis of the 19 items IQIB-ECEC scale, on the same sample data. The reliability analysis will allow us to investigate the features of the IQIB-ECEC scale as well as the items that comprise the scales. The reliability analysis process computes a variety of regularly used measures of scale reliability as well as information on the relationships between particular scale items.

The final IQIB-ECEC 19-item scale obtained a Cronbach’s alpha employed to check the internal consistency of the scale of 0.77, with a scale mean of 4.49 and a standard deviation of 0.304, a reasonable coefficient in regards to the 8-dimensionality envisaged (Ajzen, 1991; Ajzen, 2011a; Ajzen, 2011b). None of the items if dropped would have raised the internal consistency of the scale (Table 3).

**Table 3:** Item reliability statistics for the final IQIB-ECEC 19-item scale.

If item dropped					
	Mean	SD	Item-rest correlation	Cronbach’s α	McDonald’s ω
Item 1	4.73	0.472	0.3147	0.747	0.770
Item 2	4.80	0.425	0.3066	0.748	0.770
Item 5	4.65	0.617	0.0796	0.761	0.787
Item 6	4.54	0.705	0.1306	0.760	0.783
Item 7	4.33	0.789	0.3129	0.746	0.772
Item 8	4.83	0.480	0.1841	0.754	0.780
Item 10	4.59	0.676	0.2682	0.749	0.775
Item 11	4.42	0.716	0.2675	0.750	0.774
Item 13	4.82	0.447	0.2216	0.752	0.776
Item 14	4.48	0.734	0.1871	0.756	0.778
Item 15	4.67	0.580	0.1680	0.755	0.779
Item 16	4.44	0.762	0.5320	0.727	0.756
Item 17	4.31	0.822	0.4713	0.732	0.761
Item 18	4.34	0.804	0.5591	0.724	0.756
Item 20	3.49	1.170	0.4491	0.735	0.768
Item 21	3.64	1.153	0.4449	0.736	0.768
Item 22	4.57	0.648	0.4725	0.735	0.759
Item 23	4.85	0.379	0.4852	0.741	0.754
Item 24	4.84	0.382	0.4889	0.741	0.753

The IQIB-ECEC scale based on the theory of planned behavior showed similar intentional behavioral patterns to other research done in educational settings (Appendix), demonstrating the importance of attitudes and subjective norms in predicted teacher behaviors in the context of typically inclusive education (Leatherman and Niemeyer, 2005; Mahat, 2008; MacFarlane and Woolfson, 2013; Garrote et al., 2020; Desombre et al., 2021). Our research focus is on both inclusive and qualitative behaviors, a focus that has never been a topic analyzed so far, even if the SDG4.2 agenda conceptually delimitates this complex competence of preschool teachers of being both inclusive and qualitative.

Concurrent and discriminant validation was not possible due to the inexistence of valid scales for preschool teachers envisaging both inclusive and qualitative intentional behaviors based on TPB theory.

### 5.2. Network analysis

We will further analyze the meaning of the results, while conducting a network analysis (Jasp software) of the eight dimensions to better understand their relationship. Recent empirical and theoretical evaluations of social networks are discussed, with a focus on psychologic network analysis. Network analysis is a novel and promising tool for describing interactions between several variables. We estimate the relationship between all variables directly rather than attempting to reduce the structure of the variables to their shared information, as is done in latent variable modeling. A network is any system that may be represented by nodes (circles) connected by edges (lines) that indicate the strength of the connection between the nodes. Nodes represent observable variables in psychological networks, while edges show the strength of correlations between two variables, often after controlling for all other factors in the dataset (Burger et al., 2022).

We can use a regularized estimating approach instead of correlations, such as the Extended Bayesian Information Criterion Graphical Least Absolute Shrinkage and Selection Operator, or EBICglasso for short. The EBICglasso calculates partial correlations between all variables and reduces absolute weights to zero. As a result, edge weights are rather distorted, but small edge weights are reduced to zero. This hyperparameter is determined in the EBICglasso using the BIC, an information criterion that considers both model complexity and model fit.

**Figure 3:**
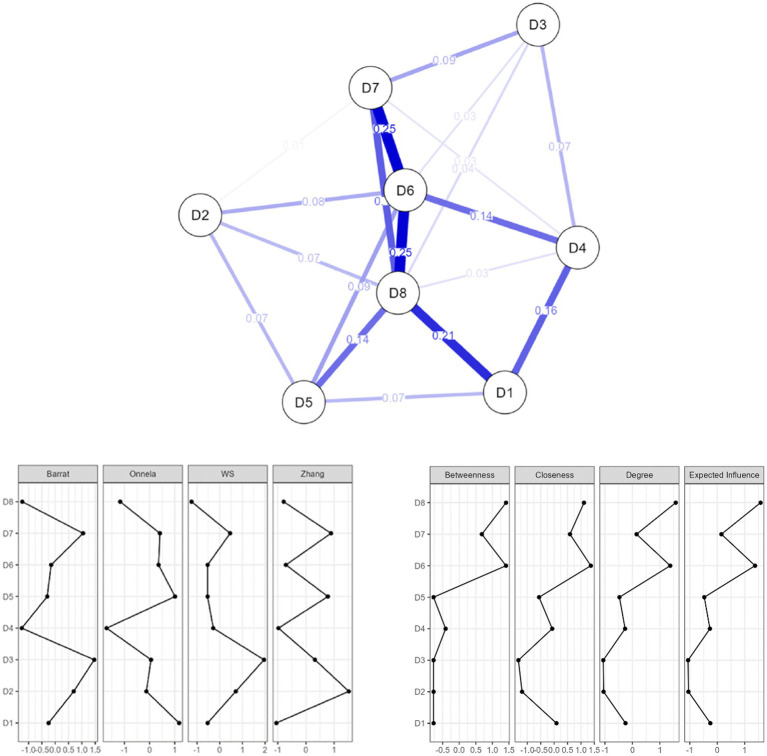
Network analysis centrality and clustering plot of the 8 dimensions of IQIB-ECEC scale.

This network has 8 nodes representing the 8 dimensions of the IQIB-ECEC 19-item scale, a maximum of 28 edges, and a sparsity value of 0.321. This score implies that the network has a low degree of sparsity and a high level of density. This sparsity rate is adequate for a network with 8 nodes. The analysis’s use of the EBICglasso estimation reduced the number of estimated edges to 19. Figure 3 is an illustration of the EBICglasso network.

Nodes represent items in psychological networks, whereas edges reflect correlations or predictive associations that may be calculated from data. In our case, each node represents one of the 8 dimensions.

The direction and strength of the connection between nodes, or in our case, dimensions, are indicated by edges. The edge may be positive, as in the case of positive covariance or correlation between the items, or it may be negative. Different colored lines to depict the edges of the graph show the polarity of the interactions: positive relationships are often colored blue or green, while negative relationships are typically colored red (Rhemtulla et al., 2016; Hevey, 2018). As shown in Figure 3, nodes are associated all positively with one another. A weighted edge changes the thickness and color density of the edge linking the nodes to show the strength of a node-to-node link: larger, denser colored lines denote stronger relationships. In a network where there are no connections between nodes, the edge may instead be unweighted and merely indicate whether a link is present or absent.

We have processed our data using R-packages: bootnet, glasso, huge, and mgm (Fruchterman and Reingold, 1991; Friedman et al., 2008; Kraeamer et al., 2009; Foygel and Drton, 2010; Epskamp et al., 2012; Epskamp, 2014; van Borkulo et al., 2014; Haslbeck and Waldorp, 2015; Zhao et al., 2015; Epskamp et al., 2016; Rhemtulla et al., 2016; Robinaugh et al., 2016; Hevey, 2018).

The study’s scope included determining the position of the network’s eight dimensions. Major centrality indicators such as strength, betweenness, closeness, and expected influence, as well as network density measures such as clustering coefficient indexes, were utilized to evaluate the connections. In order to select the most efficient node, each of these centrality measures makes a different assumption. As a result, each takes a unique strategy to making each node effective or central in a network.

As seen in Table 4, there are four centrality measures employed: betweenness, closeness, strength, and expected influence to identify highly influential nodes (Robinaugh et al., 2016).

**Table 4:** Centrality measures per variable.

Variable	Network			
	Betweenness	Closeness	Strength	Expected influence
D1	−0.773	0.086	−0.251	−0.251
D2	−0.773	−1.196	−1.050	−1.050
D3	−0.773	−1.332	−1.063	−1.063
D4	−0.409	−0.071	−0.267	−0.267
D5	−0.773	−0.561	−0.473	−0.473
D6	**1.410**	**1.370**	1.377	1.377
D7	0.682	0.590	0.150	0.150
D8	**1.410**	1.115	**1.577**	**1.577**

The highest values were bolded and discussed in text.

Nodes with a high degree of betweenness are nodes that operate as bridges between two or more clusters of nodes that are unable to communicate with one another, and they have the capacity to govern the network. The degree of closeness indicates how near one dimension is to all others. The inverse of farness, that is, the sum of the shortest distances between a node and all other nodes, is defined as the degree of closeness. This value indicates the dimension with which a dimension will have the quickest connection. Furthermore, a central node is swiftly influenced by changes in any area of the network that is close to it, and it can quickly affect changes in distant regions of the network.

Based on this finding regarding the general structure of the network, it can be said that there is a relationship between the variables and that the variables interact with each other. In the study, four types of measures were used to determine the centrality levels of the dimensions. They were betweenness, closeness, strength, and expected influence. Table 4 presents the centrality measures in detail.

Nodes having a high degree of betweenness are regarded to be in a more important position. As a result, among the variables, D6. Perceived power/Control beliefs and D8. Behavioral intention are the major variables that are highly active in the network (Table 1) and serve as a bridge between other disconnected variables (Figure 1). As a result, the total network is made up of strongly correlated variables, with D6. Perceived power/Control beliefs and D8. Behavioral intention being the two most crucial. Both dimensions 6 and 8 have both the biggest effect over the flow between all dimensions in terms of betweenness. In terms of closeness, the dimension best placed to influence the entire network most quickly is D6. Perceived power/Control beliefs. In terms of strength, the most influential dimension over its immediate neighbors is D8. Behavioral intention, and in terms of expected influence, the same dimension D8. Behavioral intention presents the most prominent characteristics in the analyzed network.

The clustering coefficient measures local cohesiveness and is defined as the fraction of connected neighbors for any vertex. These coefficients reveal how strongly the dimensions are connected with their neighbors. Clustering coefficients for the dimensions are given in Table 5.

**Table 5:** Clustering measures per variable.

Variable	Network			
	Barrat	Onnela	WS	Zhang
D1	−0.249	1.173	−0.532	−1.047
D2	0.693	−0.129	0.710	1.524
D3	1.463	0.058	1.952	0.323
D4	−1.256	−1.709	−0.284	−0.973
D5	−0.301	1.011	−0.532	0.780
D6	−0.153	0.356	−0.532	−0.709
D7	1.044	0.410	0.461	0.892
D8	−1.239	−1.170	−1.242	−0.789

A clustering coefficient is a measure of degree to which nodes in a graph tend to cluster together. Clustering coefficient of a network plays a vital role to influence the behavior of the link prediction technique (Gupta and Sardana, 2015). Clustering coefficients, which quantify the clustering propensity of the network’s dimensions, are employed to quantify the frequency of the dimensions in the network. This coefficient, which represents the frequency with which the dimensions are associated, also represents the dimension’s importance to the network. A high clustering coefficient indicates that the variables are frequently connected, while a low coefficient indicates that the linkages are rare.

## 6. Conclusion

The objective of this research was first to develop and validate a scale to assess the integrative-qualitative intentional behavior (IQIB-ECEC) of preschool teachers in order to achieve SDG4.2’s objective of ensuring that all children have access to high-quality pre-primary education and then to systemically analyze the relationship between variables with Network Analysis. Thus, the research first aimed to develop a valid and reliable scale to measure preschool teachers’ intentional integrative-qualitative behavior based on the theory of planned behavior methodology in order to further assess with Network Analysis the intentional integrative and qualitative behavioral pattern of 300 Romanian preschool teachers. Based on our previous scoping review that clearly indicated that there is a lack of assessing both qualitative and inclusive behaviors in early education (Rad et al., 2022b), we have proposed the IQIB – ECEC scale that will further analyze preschool teachers’ behavior with Network Analysis approach.

The basis for the item’s generation was the Ajzen methodology and all 24 items were adapted to qualitative-inclusive behaviors in early education for teachers. The 24 items of IQIB-ECEC were measured on a 5-point Likert scale. On a sample of 300 Romanian preschool educators, confirmatory factor analyses proved the IQIB-ECEC scale based on Ajzen’s planned behavior theory had eight subscales: actual behavior, attitudes toward the behavior, behavioral beliefs, subjective norm, normative beliefs, perceived power/control beliefs, perceived behavioral control, and behavioral intention, deleting 5 items from the final version of the scale do to negative or very low factor loadings.

Item analysis, confirmatory factor analysis, Cronbach’s alpha, and CFI, TLI, SRMR, and RMSEA fit indices were used to examine the validity and internal consistency of the IQIB-ECEC scale. The data validation demonstrated that IQIB-ECEC scale obtained an overall reliable score.

The final scale of 19 items and eight factors has very acceptable construct validity and psychometric properties and should be valuable in further investigations of integrative-qualitative intentional behaviors in preschool education toward both inclusive and qualitative early childhood education as envisaged by SDG4.2. Results indicate that IQIB-ECEC is a valid and reliable measurement for the assessment of integrative-qualitative intentional behaviors in preschool education in terms of actual behavior, behavioral beliefs, perceived power/control beliefs, perceived behavioral control, and behavioral intention.

The systemic Network Analysis approach was used in interpreting data because it is able to effectively operate at multiple levels, and it describes and makes inferences about relational properties of items, of dimensions/factors, and the entire architecture of intentional behavior.

D6. Perceived power/Control beliefs and D8. Behavioral intention are the major variables that are highly active in the network and serve as a bridge between other disconnected variables. As a result, the total network is made up of strongly correlated variables, with D6. Perceived power/Control beliefs and D8. Behavioral intention being the two most crucial. Both dimensions 6 and 8 have the biggest effect over the flow between all dimensions in terms of betweenness. In terms of closeness, the dimension best placed to influence the entire network most quickly is D6. Perceived power/Control beliefs. In terms of strength, the most influential dimension over its immediate neighbors is D8. Behavioral intention, and in terms of expected influence, the same dimension D8. Behavioral intention presents the most prominent characteristics in the analyzed network.

Behavioral beliefs connect one’s intention to the expected consequences and experiences. A behavioral belief is the perceived likelihood that a certain activity will result in a specific event or experience. Although a person may have many behavioral beliefs about any behavior, only a limited number are easily available at any given time. It is considered that the prevalent attitude toward the conduct is determined by these accessible beliefs in conjunction with the subjective values of the expected consequences and experiences. In particular, the appraisal of each outcome or experience adds to the attitude in direct proportion to the person’s subjective probability that the activity creates the desired outcome or experience.

The main conclusion of this research is that theory-grounded data can provide information required to comprehend individual perspectives and develop appropriate intervention strategies on areas of expertise and attitudes that need further instilling for the attainment of SDG4.2 goals by 2030, when developing behavioral interventions to address the increasing integrative-qualitative intentional behavior in preschool teachers from a systemically point of view.

## 7. Discussion and limitations

Several scales for measuring the degree of quality and inclusiveness in early childhood education have been reported (Soukakou, 2012; Ishimine and Tayler, 2014; Soukakou et al., 2018; Steed et al., 2022), but none approached the assessment under the theory of planned behavior framework.

This research results are consistent with previously published prominent studies. Studies show that incorporating children with disabilities in typical preschool classrooms does not result in inferior quality programs or less suitable teacher-child relationships, especially for children with mild to moderate disabilities. The findings highlight the significance of continuing education for early childhood practitioners on high-quality teacher-child interactions (Hestenes et al., 2008). Recognizing that inclusive education can take place in a variety of early childhood education programs involves taking context into account as a potential factor impacting its high-quality execution (Buysse et al., 1999; Buysse and Hollingsworth, 2009; Love and Horn, 2021).

Despite some limitation consisting of the sample characteristics, namely (1) 300 preschool teachers selected among the 700 participants in the national training program entitled Qualitative and Inclusive Early Childhood Education, (2) the planned behavior theoretical single approach of the integrative-qualitative intentional behavior in preschool education scale design, and lastly (3) the CFA used by our team that might yell that we have excluded the qualitative analysis instead of focusing exclusively on statistical measurements, this research represents one of the first studies in applying TPB to integrative-qualitative intentional behavior in preschool teachers and also a preliminary investigation of a factorial model that could be used to further asses these type of behaviors and to represent a basis for further developing behavioral change trainings for instilling integrative-qualitative intentional behavior in preschool education.

The main limitation of this research is represented by the fact that most of the items in the questionnaire have a very high mean in the results (4.5 or higher, from range 1–5, see Table 3), and with such high averages, the instrument does not effectively produce differentiations between different groups of respondents. We argue that the high item means obtained in this research is due to the sensitive aspect and the social desirability imposed by the topic, namely intentional integrative-qualitative behavior in early childhood education and care. In the majority of the cases, the 300 female preschool educators that participated in this research declared that they are willing to further adopt integrative and qualitative behaviors at work. Thus, the high averages on scale’s items represent the most important limitation of the IQIB-ECEC 19-item scale.

Future studies should focus on further making comparisons between respondents based on socio-demographic characteristics like gender, age, previous work experience, rural–urban areas, public-private institutions, and development regions.

In order to achieve the inclusive and high-quality early childhood education envisioned by SDG4.2, further research on integrative-qualitative intentional behaviors in preschool education should use the final scale, which has 19 items and eight factors, and has very acceptable construct validity and psychometric properties. Results indicate that IQIB-ECEC is a valid and reliable measurement for the assessment of integrative qualitative intentional behaviors in preschool education and the most important dimensions that impact the network of integrative-qualitative intentional behavior of Romanian preschool teachers are D6. Perceived power/Control beliefs and D8. Behavioral intention.

## 8. Implications for decision-makers

Romania’s early childhood development system is currently dealing with significant challenges. The traditional approach to kindergarten design and teaching methods must be changed in order to create new educational experiences. The pedagogy of today’s educational institutions is closely related to their design, which asks for open, adaptable, and child environments that may better support youngsters’ learning activities. We can facilitate better integrative-qualitative behaviors for the smooth achievement of SDG4.2 targets by paying close attention to the role of the preschool teacher as the primary change agent and intended recipient of the regulations and conducting a deep assessment of teachers’ perceptions of the innovative methods to early childhood education.

The primary architects of the children’s mental architecture are the preschool instructors. The preschool education system must make the transition to a child-centered architectural approach to educational design, which puts the child at the center of the design process and aims to maximize constructive interactions between children and the learning environment.

The ability of the professionals to form wholesome relationships with young children and their knowledge and abilities are the very essence of high-quality early childhood care. The alarming lack of skilled workers in the field today suggests that making significant efforts in educating, attracting, rewarding, and keeping a skilled workforce must be a key concern. Services for young children and their families should be responsibly funded with an eye on benefits against costs. Services that are inexpensive yet fall short of expectations are a waste of money.

It is both a fundamental moral obligation and a vital investment in the social and economic future of our country to address large disparities in opportunity, starting in early childhood. The study of early childhood development can offer a strong foundation for making wise decisions among competing goals and for fostering agreement on a common course of action. Such prudent decisions and concentrated dedication would be in the best interests of the safety of our society’s future. A strong foundation for future academic achievement, enhanced productivity at work, and responsible community engagement throughout adulthood is laid by policy initiatives that support nurturing relationships and a wealth of learning opportunities for young children.

The growth of all children is continually tracked through systems, allowing for the early detection of issues that require attention and the formulation of viable solutions. This can be done in the context of routine medical treatment by adequately qualified doctors, nurse practitioners, or developmental experts as well as by continued monitoring of qualified early care and education professionals.

To ensure that all children are enrolled in early intervention services, outreach efforts should be increased, so that children with developmental disabilities can learn the adaptive skills necessary to attain their full potential. Early treatments that encourage good changes in development can lay a stronger basis for subsequent attainment of higher-level talents. This emphasizes the essential necessity to detect sensory deficiencies as soon as possible after birth in order to give corrective equipment as well as the proper recovery-oriented services during the period when the fundamental brain architecture is developing.

When policymakers guarantee that all young children who are at high risk of falling behind in school participate in high-quality, evidence-based programs, the benefits are substantially greater than when only a sample of eligible children is served. It also emphasizes the need to avoid prematurely categorizing families and children who may benefit from early care as being vulnerable. The knowledge, aptitude, and interpersonal relationships skills of the teachers (Samfira and Maricuţoiu, 2021; Samfira and Paloş, 2021) reflect the quality of the early childhood services provided.

## Data availability statement

The raw data supporting the conclusions of this article will be made available by the authors, without undue reservation.

## Ethics statement

The studies involving human participants were reviewed and approved by Centrul de Cercetare Dezvoltare si Inovare in Psihologie. The patients/participants provided their written informed consent to participate in this study.

## Author contributions

GB-D: reviewing the methodology of the paper. LC: reviewing the results of the paper. GV: reviewing the conclusions of the paper. All authors contributed to the article and approved the submitted version.

## Funding

The present research’s data collection was supported by the Romanian Educational Ministry (project title: *Educație timpurie inclizivă și de calitate*), and report published by Asociația Oamenilor de Știință din România (project title: *Qualitative and Inclusive ECEC for SDG4 - a Neural Network behavioral modeling approach*).

## Conflict of interest

The authors declare that the research was conducted in the absence of any commercial or financial relationships that could be construed as a potential conflict of interest.

## Publisher’s note

All claims expressed in this article are solely those of the authors and do not necessarily represent those of their affiliated organizations, or those of the publisher, the editors and the reviewers. Any product that may be evaluated in this article, or claim that may be made by its manufacturer, is not guaranteed or endorsed by the publisher.

## Appendix

### Integrative-qualitative intentional behavior in preschool education scale (IQIB - ECEC)

**Authors:** *Dana Rad, Adela Redeș, Alina Roman, Anca Egerău, Raul Lile, Edgar Demeter, Tiberiu Dughi, Sonia Ignat, Evelina Balaș, Roxana Maier, Csaba Kiss, Vasile Mărineanu, Mușata Bocoș, Graziella Corina Bâtcă-Dumitru, Lavinia Denisia Cuc, Gabriela Vancu, Gavril Rad, Roxana Chiș.*

**Table:**

**Dimension**	**Item**
D1. Actual behavior	1. I propose daily activities in which all the children feel good.
	2. If a child is not accepted by the collective, I always find integration solutions.
D2. Attitudes toward the behavior	3. I worry about a child if they feel marginalized.
	4. By early observing children’s behavior, problematic situations can be prevented.
D3. Behavioral beliefs	5. I believe that all children should benefit from the same services.
	6. In my opinion, all children should have equal access to educational resources.
D4. Subjective norm	7. Most people close to me believe that the integration of all children into the collective is essential.
	8. Most educators consider themselves to be objective in evaluating children’s progress.
D5. Normative beliefs	9. It is necessary for the kindergarten to provide more educational resources.
	10. It would be very good for the children if the integration process is permanently monitored.
	11. Not only the kindergarten can ensure the holistic integration of the child in society.
D6. Perceived power/Control beliefs	12. It is up to me to keep all class problematic situations under control.
	13. It is in my power to ensure that no child is discriminated.
	14. It is in my knowledge that the integration of children is holistic.
D7. Perceived behavioral control	15. The decision to equally relate to all children belongs only to me.
	16. I do not need support to deal with unforeseen situations that arise in the class.
D8. Behavioral intention	17. I expect to be responsive to all the children’s needs regardless of my mood.
	18. I want to be receptive to all the needs of children regardless of the situation.
	19. I intend to be receptive to all children’s needs, regardless of context.

*Notes: The IQIB - ECEC is rated on a 1 to 5 Likert scale, where 1 represent total disagreement and 5 represents total agreement*.

*For IQIB - ECEC scale interpretation the average mean of all 19 items can further be utilized*.

*The higher the score, the higher is the integrative qualitative intentional behavior of the preschool teachers*.

## References

[ref2] Ajzen I. (1991). The theory of planned behavior. Organ. Behav. Hum. Decis. Process. 50, 179–211. doi: 10.1016/0749-5978(91)90020-T

[ref3] Ajzen I. (2011a). Design and evaluation guided by the theory of planned behavior. Soc. psychol. Eval., Guilford Publications, 74–100.

[ref4] Ajzen I. (2011b). The theory of planned behaviour: reactions and reflections. Psychol. Health 26, 1113–1127. doi: 10.1080/08870446.2011.613995, PMID: 21929476

[ref5] Ajzen I. (2020). The theory of planned behavior: frequently asked questions. Hum. Behav. Emerg. Technol. 2, 314–324. doi: 10.1002/hbe2.195

[ref6] Ajzen I. Joyce N. Sheikh S. Cote N. G. (2011). Knowledge and the prediction of behavior: the role of information accuracy in the theory of planned behavior. Basic Appl. Soc. Psychol. 33, 101–117. doi: 10.1080/01973533.2011.568834

[ref7] Arnold J. Loan-Clarke J. Coombs C. Wilkinson A. Park J. Preston D. (2006). How well can the theory of planned behavior account for occupational intentions? J. Vocat. Behav. 69, 374–390. doi: 10.1016/j.jvb.2006.07.006

[ref9] Bornschlegl M. Townshend K. Caltabiano N. (2021). Application of the theory of planned behavior to identify variables related to academic help seeking in higher education. Front. Educ. 6:738790. doi: 10.3389/feduc.2021.738790

[ref11] Brown T. A. Moore M. T. (2012). “Confirmatory factor analysis,” in Handbook of Structural Equation Modeling. ed. R. H. Hoyle (New York: The Guilford Press), 361–379.

[ref12] Burger J. Isvoranu A. M. Lunansky G. Haslbeck J. Epskamp S. Hoekstra R. H.. (2022). Reporting standards for psychological network analyses in cross-sectional data. Psychol. Methods. Advance online publication. doi: 10.1037/met0000471, PMID: 35404629

[ref13] Burns M. E. Houser M. L. Farris K. L. (2018). Theory of planned behavior in the classroom: an examination of the instructor confirmation-interaction model. High. Educ. 75, 1091–1108. doi: 10.1007/s10734-017-0187-0

[ref14] Buysse V. Hollingsworth H. L. (2009). Program quality and early childhood inclusion: recommendations for professional development. Top. Early Child. Spec. Educ. 29, 119–128. doi: 10.1177/0271121409332233

[ref15] Buysse V. Wesley P. W. Bryant D. Gardner D. (1999). Quality of early childhood programs in inclusive and noninclusive settings. Except. Child. 65, 301–314. doi: 10.1177/001440299906500302

[ref16] Cooper G. Barkatsas T. Strathdee R. (2016). “The theory of planned behaviour (TPB) in educational research using structural equation modelling (SEM),” in Global Learning in the 21st Century (Leiden: Brill), 139–162.

[ref18] Czerniak C. M. Lumpe A. T. (1996). Predictors of science fair participation using the theory of planned behavior. Sch. Sci. Math. 96, 355–361. doi: 10.1111/j.1949-8594.1996.tb15853.x

[ref19] Darker C. D. French D. P. (2009). What sense do people make of a theory of planned behaviour questionnaire? A think-aloud study. J. Health Psychol. 14, 861–871. doi: 10.1177/1359105309340983 19786512

[ref20] Desombre C. Delaval M. Jury M. (2021). Influence of social support on teachers' attitudes toward inclusive education. Front. Psychol. 12:736535. doi: 10.3389/fpsyg.2021.736535 34659050 PMC8514827

[ref21] Dunn R. Hattie J. Bowles T. (2018). Using the theory of planned behavior to explore teachers’ intentions to engage in ongoing teacher professional learning. Stud. Educ. Eval. 59, 288–294. doi: 10.1016/j.stueduc.2018.10.001

[ref23] Epskamp S. (2014). IsingSampler: sampling methods and distribution functions for the Ising model. Available at: github.com/SachaEpskamp/IsingSampler

[ref24] Epskamp S. Borsboom D. Fried E. I. (2016). Estimating psychological networks and their accuracy: A tutorial paper. arXiv:1604.08462. arXiv [preprint] 10.3758/s13428-017-0862-1 PMC5809547 28342071

[ref25] Epskamp S. Cramer A. O. Waldorp L. J. Schmittmann V. D. Borsboom D. (2012). Qgraph: network visualizations of relationships in psychometric data. J. Stat. Softw. 48, 1–18. doi: 10.18637/jss.v048.i04

[ref27] Fokkema M. Greiff S. (2017). How performing PCA and CFA on the same data equals trouble: Overfitting in the assessment of internal structure and some editorial thoughts on it. Eur. J. Psychol. Assess. 33, 399–402. doi: 10.1027/1015-5759/a000460

[ref28] Foygel R. Drton M. (2010). Extended Bayesian information criteria for Gaussian graphical models. Adv. Neural Inf. Proces. Syst. 29, 604–612. doi: 10.1016/j.tate.2012.08.006

[ref29] Francis J. J. Eccles M. P. Johnston M. Walker A. Grimshaw J. Foy R.. (2004). Constructing questionnaires based on the theory of planned behaviour. Manual Health Serv. Res. 2010, 2–12.

[ref30] Friedman J. Hastie T. Tibshirani R. (2008). Sparse inverse covariance estimation with the graphical lasso. Biostatistics 9, 432–441. doi: 10.1093/biostatistics/kxm045, PMID: 18079126 PMC3019769

[ref32] Fruchterman T. M. Reingold E. M. (1991). Graph drawing by force-directed placement. Softw. Pract. Exp. 21, 1129–1164.

[ref33] Garrote A. Felder F. Krähenmann H. Schnepel S. Sermier Dessemontet R. Moser Opitz E. (2020). Social acceptance in inclusive classrooms: the role of teacher attitudes toward inclusion and classroom management. Front. Educ. 5:582873. doi: 10.3389/feduc.2020.58287

[ref34] González S. T. López M. C. N. Marcos Y. Q. Rodríguez-Marín J. (2012). Development and validation of the theory of planned behavior questionnaire in physical activity. Span. J. Psychol. 15, 801–816. doi: 10.5209/rev_SJOP.2012.v15.n2.38892 22774454

[ref35] Greaves M. Zibarras L. D. Stride C. (2013). Using the theory of planned behavior to explore environmental behavioral intentions in the workplace. J. Environ. Psychol. 34, 109–120. doi: 10.1016/j.jenvp.2013.02.003

[ref36] Gupta A. K. Sardana N. (2015). Significance of clustering coefficient over Jaccard index. In: *2015 Eighth International Conference On Contemporary Computing (IC3)* (pp. 463–466). IEEE.

[ref37] Hadadgar A. Changiz T. Masiello I. Dehghani Z. Mirshahzadeh N. Zary N. (2016). Applicability of the theory of planned behavior in explaining the general practitioners eLearning use in continuing medical education. BMC Med. Educ. 16, 1–8. doi: 10.1186/s12909-016-0738-6 27549190 PMC4994161

[ref38] Hagger M. S. Chatzisarantis N. L. Biddle S. J. (2002). The influence of autonomous and controlling motives on physical activity intentions within the theory of planned behaviour. Br. J. Health Psychol. 7, 283–297. doi: 10.1348/135910702760213689, PMID: 12614501

[ref39] Harland P. Staats H. Wilke H. A. (1999). Explaining proenvironmental intention and behavior by personal norms and the theory of planned behavior 1. J. Appl. Soc. Psychol. 29, 2505–2528. doi: 10.1111/j.1559-1816.1999.tb00123.x

[ref40] Harrington D. (2009). Confirmatory Factor Analysis. Cambridge, Massachusetts: Oxford university press.

[ref41] Haslbeck J. Waldorp L. J. (2015). Mgm: Structure estimation for time-varying mixed graphical models in high-dimensional data. arXiv:1510.06871. arXiv [preprint].

[ref43] Heath Y. Gifford R. (2002). Extending the theory of planned behavior: predicting the use of public transportation 1. J. Appl. Soc. Psychol. 32, 2154–2189. doi: 10.1111/j.1559-1816.2002.tb02068.x

[ref44] Hestenes L. L. Cassidy D. J. Shim J. Hegde A. V. (2008). Quality in inclusive preschool classrooms. Early Educ. Dev. 19, 519–540. doi: 10.1080/10409280802230973

[ref45] Heuer A. Kolvereid L. (2014). Education in entrepreneurship and the theory of planned behaviour. Eur. J. Train. Dev. 38, 506–523. doi: 10.1108/EJTD-02-2013-0019

[ref46] Hevey D. (2018). Network analysis: a brief overview and tutorial. Health Psychol. Behav. Med. 6, 301–328. doi: 10.1080/21642850.2018.1521283, PMID: 34040834 PMC8114409

[ref47] Ishimine K. Tayler C. (2014). Assessing quality in early childhood education and care. Eur. J. Educ. 49, 272–290. doi: 10.1111/ejed.12043

[ref48] Johnson S. E. Hall A. (2005). The prediction of safe lifting behavior: an application of the theory of planned behavior. J. Safety Res. 36, 63–73. doi: 10.1016/j.jsr.2004.12.004, PMID: 15752484

[ref49] Kenny D. A. Kaniskan B. McCoach D. B. (2015). The performance of RMSEA in models with small degrees of freedom. Sociol. Methods Res. 44, 486–507. doi: 10.1177/0049124114543236

[ref50] Kenny D. A. McCoach D. B. (2003). Effect of the number of variables on measures of fit in structural equation modeling. Struct. Equ. Modeling 10, 333–351. doi: 10.1207/S15328007SEM1003_1

[ref51] Kortteisto T. Kaila M. Komulainen J. Mäntyranta T. Rissanen P. (2010). Healthcare professionals' intentions to use clinical guidelines: a survey using the theory of planned behaviour. Implement. Sci. 5, 1–10. doi: 10.1186/1748-5908-5-51 20587021 PMC2902417

[ref52] Kraeamer N. Schaeafer J. Boulesteix A.-L. (2009). Regularized estimation of large-scale gene association networks using graphical gaussian models. BMC Bioinformatics 10, 1–24. doi: 10.1186/1471-2105-10-384 19930695 PMC2808166

[ref53] Leatherman J. M. Niemeyer J. A. (2005). Teachers’ attitudes toward inclusion: factors influencing classroom practice. J. Early Childhood Teach. Educ. 26, 23–36. doi: 10.1080/10901020590918979

[ref54] Lee J. Cerreto F. A. Lee J. (2010). Theory of planned behavior and teachers' decisions regarding use of educational technology. J. Educ. Technol. Soc. 13, 152–164.

[ref55] Liu C. Sun C. K. Chang Y. C. Yang S. Y. Liu T. Yang C. C. (2021). The impact of the fear of COVID-19 on purchase behavior of dietary supplements: integration of the theory of planned behavior and the protection motivation theory. Sustainability 13:12900. doi: 10.3390/su132212900

[ref56] Love H. R. Horn E. (2021). Definition, context, quality: current issues in research examining high-quality inclusive education. Top. Early Child. Spec. Educ. 40, 204–216. doi: 10.1177/0271121419846342

[ref57] MacFarlane K. Woolfson L. M. (2013). Teacher attitudes and behavior toward the inclusion of children with social, emotional and behavioral difficulties in mainstream schools: an application of the theory of planned behavior. Teach. Teach. Educ. 29, 46–52. doi: 10.1016/j.tate.2012.08.006

[ref58] Mahat M. (2008). The development of a psychometrically-sound instrument to measure Teachers' multidimensional attitudes toward inclusive education. Int. J. Spec. Educ. 23, 82–92.

[ref59] Marsh H. W. Muthén B. Asparouhov T. Lüdtke O. Robitzsch A. Morin A. J.. (2009). Exploratory structural equation modeling, integrating CFA and EFA: application to students' evaluations of university teaching. Struct. Equ. Model. Multidiscip. J. 16, 439–476. doi: 10.1080/10705510903008220

[ref60] Newham J. J. Allan C. Leahy-Warren P. Carrick-Sen D. Alderdice F. (2016). Intentions toward physical activity and resting behavior in pregnant women: using the theory of planned behavior framework in a cross-sectional study. Birth 43, 49–57. doi: 10.1111/birt.12211, PMID: 26660944

[ref61] Opoku M. P. Cuskelly M. Pedersen S. J. Rayner C. S. (2021). Applying the theory of planned behaviour in assessments of teachers’ intentions towards practicing inclusive education: A scoping review. Eur. J. Spec. Needs Educ. 36, 577–592. doi: 10.1080/08856257.2020.1779979

[ref62] Pang S. M. Tan B. C. Lau T. C. (2021). Antecedents of consumers’ purchase intention towards organic food: integration of theory of planned behavior and protection motivation theory. Sustainability 13:5218. doi: 10.3390/su13095218

[ref63] Patterson R. R. (2001). Using the theory of planned behavior as a framework for the evaluation of a professional development workshop. Microbiol. Educ. 2, 34–41. doi: 10.1128/me.2.1.34-41.2001 23653542 PMC3633115

[ref64] Poulter D. R. McKenna F. P. (2010). Evaluating the effectiveness of a road safety education intervention for pre-drivers: an application of the theory of planned behaviour. Br. J. Educ. Psychol. 80, 163–181. doi: 10.1348/014466509X468421, PMID: 20070919

[ref65] Qi X. Ploeger A. (2019). Explaining consumers' intentions towards purchasing green food in Qingdao, China: the amendment and extension of the theory of planned behavior. Appetite 133, 414–422. doi: 10.1016/j.appet.2018.12.004, PMID: 30537527

[ref66] Rad D. Magulod G. Balas E. Roman A. Egerau A. Maier R.. (2022a). A radial basis function neural network approach to predict preschool teachers’ technology acceptance behavior. Front. Psychol. 13:880753. doi: 10.3389/fpsyg.2022.880753, PMID: 35756273 PMC9218334

[ref67] Rad D. Redeş A. Roman A. Ignat S. Lile R. Demeter E.. (2022b). Pathways to inclusive and equitable quality early childhood education for achieving SDG4 goal—a scoping review. Front. Psychol. 13:955833. doi: 10.3389/fpsyg.2022.955833 35936241 PMC9354697

[ref68] Rhemtulla M. Fried E. I. Aggen S. H. Tuerlinckx F. Kendler K. S. Borsboom D. (2016). Network analysis of substance abuse and dependence symptoms. Drug Alcohol Depend. 161, 230–237. doi: 10.1016/j.drugalcdep.2016.02.005, PMID: 26898186 PMC4861635

[ref69] Robinaugh D. J. Millner A. J. McNally R. J. (2016). Identifying highly influential nodes in the complicated grief network. J. Abnorm. Psychol. 125, 747–757. doi: 10.1037/abn0000181, PMID: 27505622 PMC5060093

[ref70] Samfira E. M. Maricuţoiu L. P. (2021). Not all perfectionists are as they are assessed: an investigation of the psychometric properties of the perfectionism inventory in the teaching profession. Front. Psychol. 12:624938. doi: 10.3389/fpsyg.2021.624938, PMID: 33643154 PMC7902758

[ref71] Samfira E. M. Paloş R. (2021). Teachers’ personality, perfectionism, and self-efficacy as predictors for coping strategies based on personal resources. Front. Psychol. 12:751930. doi: 10.3389/fpsyg.2021.751930, PMID: 34795619 PMC8593193

[ref72] Schomerus G. Matschinger H. Angermeyer M. C. (2009). Attitudes that determine willingness to seek psychiatric help for depression: a representative population survey applying the theory of planned behaviour. Psychol. Med. 39, 1855–1865. doi: 10.1017/S0033291709005832, PMID: 19379538

[ref73] Soukakou E. P. (2012). Measuring quality in inclusive preschool classrooms: development and validation of the inclusive classroom profile (ICP). Early Child Res. Q. 27, 478–488. doi: 10.1016/j.ecresq.2011.12.003

[ref74] Soukakou E. Evangelou M. Holbrooke B. (2018). Inclusive classroom profile: a pilot study of its use as a professional development tool. Int. J. Incl. Educ. 22, 1124–1135. doi: 10.1080/13603116.2017.1416188

[ref75] Stanec A. D. S. (2009). The theory of planned behavior: predicting teachers’ intentions and behavior during fitness testing. J. Teach. Phys. Educ. 28, 255–271. doi: 10.1123/jtpe.28.3.255

[ref76] Steed E. A. Rausch A. Strain P. S. Bold E. Leech N. (2022). High-quality inclusion in preschool settings: A survey of early childhood personnel. Top. Early Child. Spec. Educ. 2:027112142110639. doi: 10.1177/02711214211063921

[ref77] Sun G. Acheampong R. A. Lin H. Pun V. C. (2015). Understanding walking behavior among university students using theory of planned behavior. Int. J. Environ. Res. Public Health 12, 13794–13806. doi: 10.3390/ijerph121113794, PMID: 26516895 PMC4661615

[ref78] Suwartono C. Bintamur D. (2019). Validation of the emotion regulation questionnaire (ERQ): network analysis as an alternative of confirmatory factor analysis (CFA). ANIMA Indones. Psychol. J. 34, 115–124. doi: 10.24123/aipj.v34i3.2300

[ref79] Teo T. Zhou M. Noyes J. (2016). Teachers and technology: development of an extended theory of planned behavior. Educ. Technol. Res. Dev. 64, 1033–1052. doi: 10.1007/s11423-016-9446-5

[ref80] van Borkulo C. D. Borsboom D. Epskamp S. Blanken T. F. Boschloo L. Schoevers R. A.. (2014). A new method for constructing networks from binary data. Sci. Rep. 4, 1–10. doi: 10.1038/srep05918 PMC4118196 25082149

[ref81] Wieland A. Durach C. F. Kembro J. Treiblmaier H. (2017). Statistical and judgmental criteria for scale purification. Supply Chain Manag. 22, 321–328. doi: 10.1108/SCM-07-2016-0230

[ref82] Xia Y. Yang Y. (2019). RMSEA, CFI, and TLI in structural equation modeling with ordered categorical data: the story they tell depends on the estimation methods. Behav. Res. Methods 51, 409–428. doi: 10.3758/s13428-018-1055-2, PMID: 29869222

[ref83] Zhao T. Li X. Liu H. Roeder K. Lafferty J. Wasserman L. (2015). Huge: high-dimensional undirected graph estimation. Available at: https://CRAN.R-project.org/package=huge PMC4729207 26834510

